# Amendment and Development of National Insurance

**Published:** 1913-04-19

**Authors:** 


### NATIONAL INSURANCE ACT DEPARTMENT.

### L.—Amendment and Development of National Insurance.

A very important series of meetings convened by the newly-formed Faculty of Insurance was held on Saturday, April 5, and the results are likely to be of a more far-reaching character than the promoters can have dared to anticipate. The Faculty of Insurance is a new association that has been called into being to assist those who are administering the National Insurance Act by collecting statistics and spreading information, and by training officials through the medium of lectures and classes. The meetings referred to were attended by about 500 delegates from approved societies of all descriptions—friendly societies, collecting friendly societies, trades unions, and industrial assurance companies. The subjects discussed were all of a practical character and covered a very wide range from matters of principle to those of administrative detail. Several important resolutions were passed, but in regard to some of the subjects no such formality was adopted. One of the resolutions referred to the question of the expenses of administration, and declared that the present allowance made by the Insurance Commissioners to the approved societies is inadequate. It will be remembered that we pointed out recently that great difficulty had been experienced by many approved societies in restricting their management expenses to the limits imposed by the Insurance Commissioners, and it will not therefore come as a surprise to learn that on the first opportunity for joint deliberation a strong protest was made by those who are endeavouring to carry out the State requirements with inadequate remuneration for services rendered.

### Cost of Administration.

A suggestion was made, not for the first time, by one of the delegates, that the approved societies should be relieved of the necessity of paying postage. This is based on the plea that as the business is national the same arrangements as to franked postage should apply to the communications of the societies as to those of the Insurance Committees and Insurance Commissioners. An endeavour was made to carry this into effect some time ago, but the Postmaster-General, presumably in consultation with the Treasury, declined to make the concession. In fact a special warning was issued calling attention to the fact that all approved societies must affix stamps in the ordinary way to all postal packages except those directed to the Insurance Commissioners. The suggestion is not an unimportant one and its adoption would go a long way to reduce—if it did not actually relieve—the strain involved in keeping within the present assigned limits of expenditure. On the other hand, most—if not all—societies have private organisations as well as their State sections, and the concession would in consequence be liable to abuse. In the circumstances it is hardly likely that the concession will be granted, and it is much more probable if anything is done at all that the direct grant for expenses will be increased.

We have already pointed out in previous issues that the present cost of administration is inordinately high when it is remembered that the method of collection of contributions does not involve the weekly house-to-house visitation of the insured as is the case in industrial life assurance. The expenses are high because the Act is so complicated and the regulations so exacting. It is therefore necessary for a vigilant scrutiny to be placed on the actual expenses so that if possible improvements in the present machinery may be made with a view to removing the cause of the heavy administrative charges. An advantage possessed by the method of increasing the grant for expenses is that the exact position in regard to administrative charges could be analysed, whereas the stamp concession would obscure the issue.

The question of the administrative expenses, whilst it necessarily chiefly affects society officials, is not only their concern. It is of importance to all insured persons, because the expenses have to be met, and if the funds available are insufficient to defray the charges incurred by a society the members will have to make good the deficiency by means of a special levy.

### The Substitution of Half-Yearly Cards.

Closely related to this question, but of rather wider significance was the suggestion made by another speaker advocating the substitution of half-yearly for the present quarterly cards. Quarterly cards were a necessity at the inception of the scheme, for until the first cards had been collected the societies did not know definitely the extent of their members, and the cards had to be examined and listed, so that the necessary credit might be obtained in readiness for the payment of claims at the close of the second quarter. This phase of the scheme has now passed away, and the suggestion has much to commend it from the point of view of saving of labour to society officials, but it has the disadvantage that if a card should be lost or defaced towards the end of the half-year the loss to the insured person would be much more serious than in the case of a quarterly card. Then, again, experience has shown that many of the cards have already become disagreeable to handle at the end of a quarter.

### Life Assurance Indicated.

It will probably be agreed that the main interest in the proceedings centred in the speech of Mr. C. F. G. Masterman after the inaugural dinner. As Chairman of the Joint Committee of the Insurance Commissioners he had much to tell his audience in reference to the work that had already been accomplished, and he was warm in his praise of the support that had been accorded to the Commissioners by society officials generally. It was not, however, as a report of past work that his utterance was noteworthy, but as an indication of future developments.

The first real piece of news was that, as noted in our issue of last week, the Chancellor of the Exchequer and he would this year introduce an amending Bill, and the assurance which he gave to those present that "he would endeavour to gather any suggestion aimed at the one question as to how after an experience of twelve months it will be possible to reduce the work of those who are administering the Act or to make it still more fair and welcome to the industrial population of the country" will give general satisfaction. This statement, emanating from such authority, makes it encumbent upon all those who have been endeavouring to further the cause of National Insurance on sound lines to frame the schemes for amendment which they have evolved and to submit them without delay to the notice of the Chancellor and the Parliamentary Secretary to the Treasury.

Having indicated that amendment of the Act is under consideration Mr. Masterman made the further statement that it may be in future that life assurance will be incorporated in the scope of National Insurance, and that further powers may be obtained with the object of extending the principle of health insurance to children. It is probable that Mr. Masterman was aware when he spoke that a private member, Mr. A. C. Edwards, had already prepared a Bill for presentation to Parliament for adding life assurance to sickness insurance. This Bill was given a first reading on Wednesday, April 9, but we doubt very much whether it will pass any of the further stages of Parliamentary procedure. The Bill would require a contribution of 1d. a week in addition to the present contributions, and the stamps now being affixed to the Insurance cards would be increased by that amount. According to the suggestion of the author the whole of this additional contribution would have to be borne by the employer.

Such a scheme is bound to meet with opposition. In the first place it is not clear that life assurance is a national requirement. The Bill itself shows evidence of this, for it is laid down by the author that existing assurances are not to be displaced, and that the object of the measure is to increase the assurance protection where policies are already in existence. The fact is that the Industrial Assurance Companies and Collecting Friendly Societies have by private enterprise perfected their organisations to such a degree of efficiency that it is only a small minority of the industrial population which has not already made provision by voluntary thrift against the risk of early death. There is nothing like the same necessity for national assurance as there was for National Insurance against sickness. In these circumstances it will not only be the Industrial Life Offices and Collecting Friendly Societies, whose business would stand to be adversely affected, that will show opposition to the Bill, but the majority of members of Parliament may be expected to offer resistance to a proposal, the object of which is to provide something that is unnecessary. It is also fairly evident that the particular scheme of the Bill by which the whole cost would be thrown on the employers will meet with determined opposition from employers of labour throughout the country. The charge per workman of 1d. a week is small, it is true, but where a large number of hands are employed the aggregate sum would be considerable. Thus on the basis of 1,000 workmen the charge would amount to over £200 per annum, and it is clear that this would go to reduce profits without any corresponding gain to the employer. In the case of health insurance it has been contended that the employer receives valuable consideration for his contributions in the shape of more efficient service from his workpeople, but for benefits payable on death there can be no such advantage. The Bill now before Parliament can therefore have little chance of success, and it may be that the only object of its introduction was to ascertain the feeling of the House as to the development of National Insurance on such lines.

Life assurance was specifically excluded from the scope of the present Act, which definitely lays down that no part of any surplus that may be disclosed on the valuation of an approved society may be applied in the provision of benefits payable on death. This provision was, in fact, included to protect the Industrial Assurance Companies and Collecting Friendly Societies, and was an essential condition of their taking part in the administration of the Act. It was admitted by Mr. Masterman that the approved societies formed in connection with these organisations had rendered most efficient help in the administration of the Act during the past twelve months, and it is much to be regretted in the public interest if the Government should break faith with this important class of society. The suggestion, to say the least, is disquieting, and developments will be awaited with interest.

### Answers to Correspondents.

"JOHN BULL" writes: "In an imaginary case of a nurse being ill in hospital, at the end of three weeks she is convalescent, but not likely to be fit for work for three or four weeks. She is then sent off for her annual month's holiday. What is her position with regard to Insurance benefit? From hospital point of view she is on holiday. From an employee's point of view she is unfit for work through illness. Is she due for Insurance benefit?"

This is an imaginary case, and therefore cannot be definitely answered. The whole facts surrounding an actual case would have to be taken into consideration. The most important point is that any nurse who is so required to take her holiday during convalescence from illness should communicate with her society before she goes away. If the necessary doctor's certificate can be produced stating that she is incapable of work and the society of which she is a member is satisfied as to the genuineness of the claim, there is no reason why sickness benefit should not be paid. Unless the claim is *bonâ-fide* in all respects, the society would most certainly not admit liability.

